# Rituximab Plus Chemotherapy Provides No Clinical Benefit in a Peripheral T-Cell Lymphoma not Otherwise Specified with Aberrant Expression of CD20 and CD79a: A Case Report and Review of the Literature

**DOI:** 10.3390/diagnostics10060341

**Published:** 2020-05-26

**Authors:** Alessandro Mangogna, Maria Christina Cox, Luigi Ruco, Gianluca Lopez, Beatrice Belmonte, Arianna Di Napoli

**Affiliations:** 1 Pathology Unit, Clinical Department of Medical, Surgical and Health Science, University of Trieste, Cattinara Hospital, 34149 Trieste, Italy; alessandro.mangogna@studenti.units.it; 2 Haematology Unit, Sant’Andrea Hospital, Sapienza University, 00189 Rome, Italy; chrisscox@gmail.com; 3 Haematology Department, King’s College Hospital NHS Foundation Trust, London SE5 9RS, UK; 4 Pathology Unit, Department of Clinical and Molecular Medicine, Sant’Andrea Hospital, Sapienza University, 00189 Rome, Italy; luigi.ruco@uniroma1.it (L.R.); gianluca.lopez10@gmail.com (G.L.); 5 Tumor Immunology Unit, Human Pathology Section, Department of Health Sciences, University of Palermo, 90134 Palermo, Italy; beatrice.belmonte@unipa.it

**Keywords:** peripheral T-cell lymphoma, B-cell antigens, CD20, CD79a, rituximab

## Abstract

Peripheral T-cell lymphoma, not otherwise specified (PTCL-NOS) is the most common entity of mature T-cell neoplasms. PTCL-NOS generally has an aggressive behavior and is often refractory to standard therapy. Only a few cases of PTCL with aberrant expression of B-cell antigens have been reported so far. This phenotypic aberrancy may lead to misdiagnosis as B-cell non-Hodgkin lymphomas and eventual inappropriate patient management, whereas in an accurately diagnosed PTCL, the presence of CD20 may appear as an appealing therapeutic target. In this setting, response to anti-CD20 monoclonal antibody in combination with chemotherapy has been poorly explored. We describe the case of a 59-year-old male diagnosed by a pathological and molecular approach as PTCL-NOS with aberrant co-expression of the B-cell antigens CD20 and CD79a, which proved non-responsive to the addition of rituximab to standard polychemotherapy. This case highlights that the presence of CD20 in PTCL may be misleading in the diagnosis and also act as a lure for the clinician to adopt a rituximab-based treatment, the effectiveness of which is undefined as the molecular mechanisms underlying B-cell marker expression in PTCL.

## 1. Introduction

The term peripheral T-cell lymphoma, not otherwise specified (PTCL-NOS) encompasses a heterogeneous group of nodal and extranodal T-cell neoplasms derived from peripheral T lymphocytes exhibiting a broad cytological and phenotypic heterogeneity [1]. PTLC-NOS typically occur in adults and account for about 25% of all PTCL, which represent over 15% of all non-Hodgkin lymphomas in Europe and United States, being more common in Eastern countries [2]. PTCL-NOS frequently shows a defective T-cell phenotype with loss of CD5 and CD7 antigen expression and a prevalent CD4+/CD8- phenotype in nodal cases. CD4/CD8 double-positivity or double-negativity and expression of CD8, CD56, CD30, and cytotoxic granule can also be seen [3,4]. T-cell lymphomas usually do not express B-cell markers; nevertheless, a single B-cell marker expression, such as CD79a or CD20, has been occasionally reported in T-cell lymphoblastic lymphomas and mature T-cell lymphomas [5,6,7,8,9,10,11,12,13]. By contrast, only few cases of PTCL with aberrant expression of two or more B-cell antigens have been documented [14,15,16]. These cases expressed T-cell-associated antigens and had a clonal T-cell receptor (TCR) gamma gene (*TRG*) rearrangement without any evidence of monoclonal rearrangements of the immunoglobulin heavy- and light-chain genes (*IGH, IGK*) [14,15,16]. Nevertheless, some cases of primary T-cell lymphoma with co-expression of B-cell markers and clonal gene rearrangement for both *TRG* and *IGH* have been recently reported [17,18]. Due to its rarity, little is known about this subtype of disease, particularly regarding its treatment and prognosis, with only a few cases treated with anti-CD20 therapy alone or in combination with chemotherapy (Table 1) [9,16,17,19,20,21,22,23,24,25,26,27]. In the present report, we describe a case of PTCL-NOS characterized by concomitant strong expression of CD20 and CD79a, in which treatment with rituximab plus dexamethasone, cisplatin and cytosine arabinoside (R-DHAP) and rituximab plus gemcitabine and oxaliplatin (R-GEMOX) were not effective. 

## 2. Case Presentation

### 2.1. Clinical Data

A 59-year-old male was admitted to our Hematopathology Unit of the Sant’Andrea Hospital of Rome for fever, night sweats, and weight loss. The Eastern Cooperative Oncology Group performance status was 2. Physical examination revealed cervical, axillary, and inguinal lymphadenopathy and was negative for cutaneous rash. Whole body computed tomography showed abnormal enlargement of several superficial and deep lymph nodes (maximum diameter, 9 cm) along with splenomegaly (longitudinal diameter, 20 cm). Laboratory data showed polyclonal hypergammaglobulinemia and elevated LDH, and serologic studies were negative for HIV, HCV, and HBV. Contrast-enhanced magnetic resonance imaging of the brain was negative. The patient underwent left inguinal lymph node and iliac crest bone marrow biopsies. Both the international prognostic index (IPI) and the peripheral T-cell lymphoma scores were high. The patient provided written consent for the use of its tissue for research purposes and for publication of his disease and clinical course (Ethical Committee of Sant’Andrea Hospital/University “Sapienza” of Rome (EC n. 981/2012, 26 November 2012).

### 2.2. Pathological and Molecular Findings

**Figure 1:**
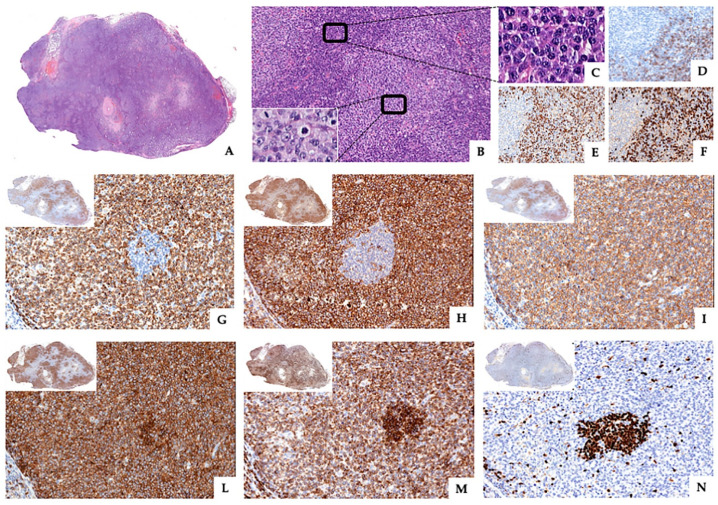
Lymph node architecture effaced by a pseudonodular infiltrate (**A** ×7, hematoxylin and eosin, H&E) of medium/large-sized atypical lymphoid cells (**B** ×100, lower inset ×400, H&E) clustered in large sheets separated by aggregates of polytypic plasma cells (**C** ×400, H&E) expressing CD138 (**D** ×400), kappa (**E** ×400), and lambda (**F** ×400) immunoglobulin light chains. Neoplastic cells stained for CD3 (**G** ×200), CD4 (**H** ×200), and CD5 (**I** ×200), and B-cell-associated antigens CD20 (**L** ×200) and CD79a (**M** ×200). Conversely, PAX5 was not expressed by the tumor cells but only by the small reactive B lymphocytes (**N** ×200).

The histological examination of the inguinal lymph node biopsy (3 cm in diameter) showed effacement of the nodal architecture due to a diffuse infiltration of medium- and large-sized lymphoid cells with round vesicular nuclei, prominent nucleoli and abundant clear cytoplasm, and frequent mitotic figures. Numerous plasma cells, scattered small lymphocytes, and epithelioid histiocytes were observed between the tumor nodules. Rare regressed germinal centers were also detectable throughout the lymph node (Figure 1A,B). 

**Figure 2:**
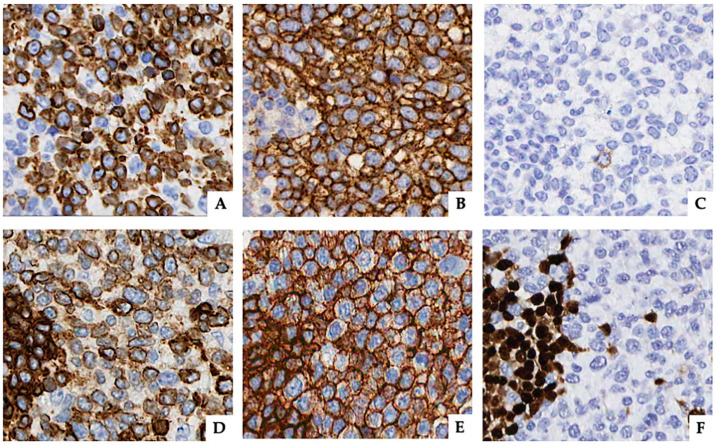
Pattern of immunohistochemical expression of different markers on neoplastic lymphocytes. Cytoplasmic and membrane staining were observed for CD3 (**A**) and CD79a (**D**). Membrane positivity was more evident for CD4 (**B**) and CD20 (**E**). PAX5 stained only nuclei of B cells (**F**), whereas < 1% of tumor cells were CD30+ (**C**) (original magnification **A**, **B**, **C**, **D**, **E**, and **F** ×400).

Immunophenotypic analysis revealed that tumor cells were positive for CD3, CD4, CD5 (Figure 1G–I and Figure 2A,B), and TIA-1 and negative for CD8, PD-1, CD21, CD23, CD10, CD56, S100, ALK, TdT, CD34, myeloperoxidase, and granzyme B (data not shown). CD30 staining showed very rare positive cells (<1% of the mononuclear cells) (Figure 2C). Of note, neoplastic cells also showed uniform, strong, and diffuse expression of the B-cell-associated antigens CD20 and CD79a (Figure 1L,M and Figure 2D,E) but were negative for PAX5 (Figure 1N and Figure 2F). Ki-67 staining revealed a high proliferative rate (~70%). Epstein–Barr virus (EBV) infection was ruled out by negativity for both in situ hybridization for EBV-encoded RNA transcripts and immunohistochemistry for EBV latent membrane protein 1. An abundant polytypical reactive plasmacytosis, positive for CD138 and both κ and λ immunoglobulin light chains, was also present (Figure 1C–F). 

**Figure 3:**
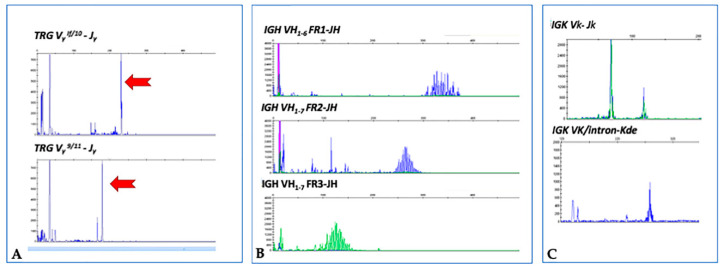
Gene scan analyses showing a clonal (red arrows) T-cell receptor gamma gene (*TRG*) (**A**) and polyclonal immunoglobulin heavy chain (*IGH*) (**B**) and light chain (*IGK*) (**C**) gene rearrangements.

Due to the concomitant expression of T- and B-cell-associated antigens, assessment of both T- and B-cell clonality was performed on the DNA extracted from the whole lymph node. Multiplex polymerase chain reaction (PCR) approaches based on the BIOMED-2 protocol were applied using the TCR gamma gene (*TRG)* rearrangement molecular analysis kit (Master Diagnostica, Granada, Spain) and the IdentiClone immunoglobulin heavy chain (*IGH*) and immunoglobulin kappa light chain (*IGK*) gene clonality kits (Invivoscribe, San Diego, CA, USA) as previously described [28]. The fluorochrome-labeled PCR products of rearranged genes were then visualized by high-resolution fragment analysis on a 3130 Genetic Analyzer (Applied Biosystems, Foster City, CA, USA) using the GeneMapper program. The analyses showed a clonal rearrangement of the *TRG* gene, given by the presence of a high single peak due to PCR products of identical size, and a polyclonal rearrangement of both the *IGH* and *IGK* genes represented by a Gaussian distribution of many different PCR products (Figure 3). 

Based on the morphological, immunohistochemical, and molecular findings, the disease was diagnosed as PTCL-NOS with aberrant co-expression of CD20 and CD79a associated with a polyclonal reactive plasmacytosis. 

**Figure 4:**
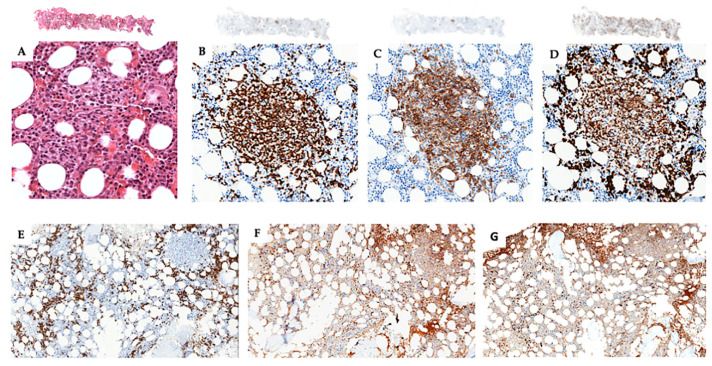
Bone marrow trephine biopsy showing focal nodular infiltration (**A** H&E) of CD3+ (**B**), CD20+ (**C**), CD79a+ (**D**) neoplastic T cells associated with a polytypical reactive CD138+ plasmacytosis (**E**) with polytypical expression of kappa (**F**) and lambda (**G**) light chains (original magnification **A**, **B**, **C**, and **D** ×200; **E**, **F**, and **G** ×100; insets ×5).

The bone marrow trephine biopsy showed a hypercellular bone marrow due to a multifocal nodular neoplastic infiltration associated with polytypical plasmacytosis (Figure 3). Of note, in the tumor nodules infiltrating the bone marrow, the neoplastic T cells still maintained the aberrant expression of both CD20 and CD79a (Figure 4). 

### 2.3. Therapeutic Strategies

The patient was treated with six cycles of CHOEP-14 (cyclophosphamide, doxorubicin, vincristine, etoposide, and prednisone). However, his condition progressed shortly after the last cycle. As brentuximab was not allowed for this CD30-negative case, R-DHAP was started, but after two cycles the patient was not responding and underwent R-GEMOX, which he continued for three cycles until clinical progression. The patient died 8 months after diagnosis.

## 3. Discussion

As implied by its name, PTCL-NOS is often a diagnosis of exclusion due to its remarkable cytological and phenotypic heterogeneity. Advanced stage is common, and the clinical outcome is unfavorable with poor response to treatments [3]. Contrary to precursor T-cell lymphoblastic leukemia/lymphoma [6,8], the expression of B-cell markers in PTCL is usually uncommon [5,6,7,8,9,10,11,12,13,14,15,16,17,18]. We reported a case of PTCL expressing both CD20 and CD79a. 

CD20 is a transmembrane calcium channel that is crucial for B-cell activation, proliferation, and differentiation [29]. It is expressed from early pre-B-cell development before the expression of surface immunoglobulin, and it is lost during terminal B-cell differentiation into plasma cells [29]. Nevertheless, it is not a perfect B-cell marker because a small number of T cells are weakly (dim) CD20 positive in disease-free individuals. In particular, two thirds of the CD20^dim^ T cells are CD8 positive, while one third express CD4. Usually, these T cells express TCR-γδ [30,31]. A number of speculations have been advanced regarding the origin of CD20 expression in T-cell lymphomas. The first possibility is that these lymphomas may represent a neoplastic transformation of a normal T-cell subset [5]. The second possibility is that the expression of CD20 could be the result of T-cell activation [7]. Indeed, it has been demonstrated that in monkey lymph nodes, T cells transiently express CD20 mRNA and weakly surface CD20 after in vivo activation with interleukin-2 and mitogen [32]. The third possible explanation for CD20 acquisition may be trogocytosis [12]. In this process, upon interaction with surrounding cells, lymphoid cells capture plasma membrane proteins from the interacting cell to express them on their own surface. This may result in the acquisition of “lineage-foreign” antigens [33]. An alternative concept is that CD20 expression simply reflects aberrant expression by the tumor population [11]. 

CD79a forms a disulfide-linked heterodimer with CD79b constituting the CD79 protein. CD79a is expressed in developing B lymphocytes before CD79b and CD19 on CD10+/CD34+/TdT+ B-cell precursors. It is found in the cytoplasm of pro-B cells before *IGH* gene rearrangement, and its expression is extinguished in terminally differentiated plasma cells [34]. Because CD79a is part of the B-cell receptor, most T-cell neoplasms do not express CD79a proteins. Nevertheless, the specificity of CD79a for B-cell neoplasms has been challenged since CD79a has been reported in up to 40% cases of precursor T-cell lymphoblastic lymphoma but very rarely in mature T-cell lymphoma [6,8,14,15,16,18]. In addition, lineage infidelity may be demonstrated in the presence of immunodeficiency and infection with EBV [35]. In our cases, the patients did not show any evidence of immunodeficiency or signs of EBV infection. 

Other mechanisms proposed for the expression of B-cell antigens on neoplastic T cells included dysregulation of the expression of transcription factors required to maintain B- or T-cell lineage commitment after the hematopoietic stem cell stage. Loss or ectopic expression may allow partial reprogramming of B- or T-cells [36,37,38,39]. For example, it has been shown that PAX-5 induces the transcription of CD19 and CD79a in B cells [40]. In vitro, in the absence of PAX-5 and under the stimulation with different cytokines, pro-B-cells acquire plasticity differentiating into macrophages, dendritic cells, granulocytes, NK cells, and T cells [39]. Moreover, PAX-5 inactivation in mature B cells induces down-regulation of several B-cell antigens and B-cell-specific genes [41]. Lazzi et al. found aberrant methylation of *PAX-5* α and β promoters in 3 PTCL-NOS expressing CD20 and CD79a but not PAX-5 [18]. Of note, PAX-5 has been found to be negative in many CD20-positive PTCL-NOS (Table 1). Similarly, in our case, the neoplastic T-cell clone did not express PAX-5, suggesting a high degree of transcriptional dysregulation. 

Little is known about the effect of B-cell antigen expression in PTCL on prognosis. Hu et al. reported that 12 Chinese patients with CD20-positive PTCL were clinically heterogeneous with a subset of patients associated with high IPI score and more aggressive clinical course [42]. Among the eight CD20+ PTCL patients with clinical follow-up reported by Rahemtullah et al., five cases with either stage I or IV disease behaved aggressively [9]. Regarding the rituximab treatment, among 13 PTCL treated with rituximab in combination with chemotherapy as first-line treatment (Table 1) 2/13 (15%) attained complete remission, and 3/13 (23%) showed stable disease, while the remaining 8/13 (62%) were refractory (i.e., partial remission in 4/8 and no response or progression in 4/18 respectively). Kakinoki et al. reviewing 9 PTCL cases from the literature found that rituximab-based chemotherapy was more effective in those showing high expression of CD20 (overall response rate 100% in 3/9 cases) compared with those with a dimmer or focal CD20 expression (overall response rate 33.3% in 6/9 cases) [19]. In our case, who was treated with R-chemotherapy as second line, tumor cells showed a strong and diffuse membrane staining for CD20; nonetheless, the disease was refractory to the addition of rituximab to two different chemotherapy regimens. 

Moreover, Hirata et al. showed the loss of CD20 expression on the neoplastic T cells infiltrating the bone marrow after rituximab therapy in a patient with CD20-positive PTCL [22]. A lack of CD20 after rituximab treatment occurred more frequently in CD20-positive PTCL (5/11 reviewed cases; 45.5%) than in B-cell lymphoma (26.3 %) [21,23], suggesting that the CD20 expression in T-cell lymphoma is unstable and may be less suitable for rituximab treatment. In our case, the lymph node and bone marrow were infiltrated by tumor cells, showing intense staining for both CD20 and CD79a. However, post-rituximab biopsies were not performed precluding CD20 expression re-assessment to challenge the hypothesis of CD20 instability in PTCL. 

In B-cell malignancies, development of resistance to rituximab has been found to be associated with different mechanisms, including the loss of CD20 expression by tumor cells. A truncated CD20 splice variant, called ΔCD20, has been detected in Raji and Ramos B-cell lines that were rendered insensitive to rituximab through repeated exposure to escalating doses of the drug [43]. The alternative splicing leads to a coded protein lacking the extracellular domain (including the RTX epitope sequence) without affecting the functions of the inner cytoplasmic tail, which includes phosphorylation sites as well as domains necessary for reorganization into lipid rafts. Another study has assigned the lower additional band identified by Western blot using an anti-CD20 antibody to a partial digestion product of the CD20 protein, suggesting that CD20 expression may be regulated by the ubiquitin–proteasome system [44]. In addition, the ΔCD20 protein may also interact with the wild-type CD20 protein modulating CD20 reorganization within the lipid raft and contributing to the development of rituximab resistance. Other mechanisms involved in the loss of CD20 expression include deletions/mutations within the C-term region of the CD20 [45], and monocyte-mediated shaving of the CD20/rituximab complex from the B-cell surface (i.e., antigenic modulation), which may occur when effector mechanisms, such as antibody-dependent cellular cytotoxicity (ADCC) have been saturated [46,47]. Of note, B-cell clones grown to be resistant to rituximab also show resistance to cytotoxic chemotherapy, suggesting underlying common mechanisms [48]. We reported a PTCL-NOS with aberrant co-expression of CD20 and CD79a characterized by a very poor prognosis, and no benefit from the addition of rituximab to two different chemotherapy schedules. It might also be possible that rituximab-mediated ADCC was impaired in our PTCL due to an immune deficiency state related to the neoplastic nature of the T-cell clone. Accordingly, in a clinical trial, the addition of rituximab to conventional chemotherapy in patients with angioimmunoblastic T-cell lymphoma and CD20+ EBV-infected large B-blasts, showed no clear benefit [49]. 

Although the molecular mechanisms driving B-cell marker expression and their function in PTCL are still unclear, the presence of CD20 seem to be an appealing therapeutic target worth of being exploited. However, rituximab has not shown a promising activity so far; indeed, only a minority of the patients reported to date (Table 1) achieved complete remission following R-chemotherapy. Recently, it was shown that in PTCL interim, FDG-PET is a valuable tool for ruling out poor responders to first-line therapy. Hopefully, this may aid in addressing PTCL to alternative approaches before completing chemotherapy if valuable options are available [50]. Brentuximab vedotin is an important adjunct to chemotherapy for all CD30+ PTCL. However, the lack of co-expression of CD30 in our as well as in other CD20+ PTCLs [9,16,17,22,24,25,27] had limited its use in this subset of PTCL. Although the expression of more than one B-cell marker in PTCL is uncommon, the activity of polatuzumab may be investigated in CD79b-positive cases. Other new agents, such as pralatrexate [51], histone deacetylases inhibitors [52], demethylation agents [53], tyrosine kinase inhibitors [54], proteasome inhibitors [52], and monoclonal antibodies as anti-CD47 [55], have shown activity in relapsed/refractory PTCL. Remarkably, patients responding to novel drugs may have a lasting remission, while the experimentation of active combinations of targeted molecules holds great expectations [56]. Patients who respond to second-line therapies, if suitable, should be addressed to allotransplant [57]. Lastly, as the majority of patients with CD20+ PTCL were ≥70 years old, the all-oral metronomic DEVEC (Deltacortene®, etoposide, vinorelbine, cyclophosphamide) schedule may also be considered as a convenient approach for elderly and vulnerable patients [58].

## 4. Conclusions

The case herein reported emphasize the necessity to apply gene rearrangement studies to unveil the actual lineage of neoplastic clones phenotypically expressing aberrant markers. The expression of both CD20 and CD79a may lead to misdiagnosis a PTCL-NOS as a large B-cell lymphoma with possible inappropriate patient management. Even though the presence of CD20 may suggest the addition of rituximab to chemotherapy, it has not shown a promising activity so far. Other drugs are therefore still needed to improve the survival of PTCL patients.

## Figures and Tables

**Table 1:** Published cases of CD20-positive peripheral T-cell lymphoma, not otherwise specified (PTCL-NOS) treated with rituximab alone or with chemotherapy.

Reference	Age (yr)/Sex	Clinical Stage	T-cell Antigens	B-cell Antigens	CD30 Antigen Expression	Molecular Findings	Therapy	Response
Magro et al. [17]	65/F	I	CD3+, CD5+, CD2+, CD4+/-, CD8+	CD20+ variable, CD79a-, CD22-	-	*TRB* clonal *IGH* clonal	R	Progressive disease
Buckner et al. [24]	84/M	III	CD3+, CD5+, CD7-, CD4+,	CD20+ variable	-	*TRG* clonal	R-CHOP	Progressive disease
Rahemtullah et al. [9] Patient 2	77/M	II	CD3+, CD5+, CD2+, CD7-, CD4+, CD8-	CD20+dim, CD79a-, PAX5-	-	*TRG* clonal	R plus chemotherapy including anthracycline	Alive at 4 months of treatment
Rahemtullah et al. [9] Patient 4	36/F	III	CD3+, CD5+, CD2+, CD7+, CD4+, CD8-	CD20+dim, CD79a-, CD19-, CD22-	ND	*TRG* and *TRB* clonal *IGH* and *IGK* polyclonal	R plus chemotherapy including anthracycline	Partial remission
Rahemtullah et al. [9] Patient 5	75/M	IV	CD3+, CD5-, CD2-, CD7+, CD4-, CD8-	CD20+strong, CD79a+, CD19+ PAX5-	+ (rare positive cells)	*TRG* and *TRB* clonal *IGH* polyclonal	R plus chemotherapy including anthracycline	Partial remission
Makita et al. [26] *	59/M	IV	CD3+, CD5+, CD7+, CD4-, CD8-, GrB+, TIA1+	CD20+strong, CD79a-, PAX5-	ND	*TRB* clonal *IGH* polyclonal	R-CHOP	Progressive disease
Hirata et al. [22]	74/M	III	CD3+, CD5+, CD2+, CD7+, CD4+, CD8-	CD20+ variable, CD79a-, CD19-, CD22-	-	*TRG* clonal *IGH* polyclonal	R	Stable disease
Cumiskey et al. [25]	84/M	III	CD3+, CD5+, CD4+, CD8-	CD20+ strong, CD79a-	-	TCR (gene not specified) clonal *IGH* polyclonal	R-CEOP	Complete remission
Matnani et al. [16]	75/M	IV	CD3+, CD5+/-, CD7+/-, CD4-, CD8-	CD20+ variable, CD19+, CD79a-	-	*TRG* monoclonal *IGH* polyclonal	R-CHOP	Complete remission
Kamata et al. [27]	83/F	IV	CD3+, CD5+, CD4+, CD8-	CD20+ variable, CD79a-, PAX5-	-	*TRG* clonal *IGH* polyclonal	R-CHOP	Partial remission
Kakinoki et al. [19]	44/M	IE	CD3+, CD5+, CD7+, CD4-, CD8-	CD20+, CD79a- variable, PAX5-	ND	*TRG and TRB* clonal *IGH* polyclonal	R-CHOP	Stable disease
Teshima et al. [21] *	79/M	III	n/a	CD20+	ND	TCR (gene not specified) clonal *IGH* polyclonal	R-CHOP	Partial remission
Shao et al. [20]	65/M	III	CD3+	CD20+, PAX5-	ND	TCR (gene not specified) clonal	R-pGEMOX	Partial remission
Mangogna et al. (our case)	59/M	IV	CD3+, CD5+, CD4+, CD8-, TIA1+, GrB-	CD20+, CD79a+, PAX5-	+ (rare positive cells < 1%)	*TRG* clonal *IGH* polyclonal *IGK* polyclonal	R-DHAP R-GEMOX	Progressive disease

Abbreviations: GrB = granzyme B; ND = not determined; TCR = T-cell receptor; *TRG* = T-cell receptor gamma gene; *TRB* = T-cell receptor beta gene; *IGH* = immunoglobulin heavy chain gene; *IGK* = immunoglobulin kappa light chain gene; R = rituximab; R-CHOP = rituximab plus cyclophosphamide, doxorubicin, vincristine, and prednisolone; R-(p)GEMOX = rituximab plus (L-asparaginase), gemcitabine and oxaliplatin; R-CEOP = rituximab plus cyclophosphamide, etoposide, vincristine, and prednisolone; R-DHAP = rituximab plus dexamethasone, cisplatin, and cytosine arabinoside; n/a = not available. *: Article in Japanese, only abstract available.

## Abbreviations

CHOEP-14	cyclophosphamide, doxorubicin, vincristine, etoposide, prednisone
EBV	Epstein–Barr virus
*IGH*	immunoglobulin heavy-chain gene
*IGK*	immunoglobulin light-chain gene
IPI	international prognostic index
PCR	polymerase chain reaction
PTLC-NOS	peripheral T-cell lymphoma, not otherwise specified
R	rituximab
R-DHAP	rituximab plus dexamethasone, cisplatin and cytosine arabinoside
R-(p)GEMOX	rituximab plus (L-asparaginase), gemcitabine and oxaliplatin
TCR	T-cell receptor
*TRG*	T-cell receptor gamma gene
